# Honeybee sting of the sclera: occular features, treatment, outcome and presumed pathogenesis

**DOI:** 10.11604/pamj.2014.17.30.3297

**Published:** 2014-01-17

**Authors:** Michaeline Asuquo Isawumi, Mustapha B Hassan

**Affiliations:** 1 Osun State University, PMB 4494 Osogbo, Nigeria

**Keywords:** Sclera bee sting, features, treatment, outcome, presumed pathogenesis

## Abstract

Ocular bee sting injury has caused several reactions in the eye but has rarely been reported among local African farmers, and Nigerians in particular. This case seeks to report the first ocular and external eye reactions following a honey bee sting of the eye through the sclera, highlighting the treatment and outcome. Oral interview, clinical examination and external photographs were used to obtain and document findings. Medical treatment was instituted as soon as subject presented. There was complete inflammatory resolution within a week, normal vision and no evidence of stinger migration after four weeks of follow up. The wound site healed with ciliary staphyloma. The role of physical properties, immunological and genetics interplay and the presumed pathogenesis is further discussed. Health education on early presentation and avoidance of harmful traditional eye medications should be promoted among the farming populations in our communities, in order to prevent blinding complications

## Introduction

The honey bee and wasps belong to the hymenoptera specie of insects. The stinger is made of stylet-wrapped double lancets, with a saw-like tip and an upper bulb.[1] The saw-like tip gives it the ability to remain embedded in tissues and complete removal difficult without expertise. Injury can results from the bee stinger or venom. Studies have documented that its toxin can stimulate severe allergic reactions in the body, affecting various organs like the kidney, heart, nervous systems and the eye.[2] The ocular reactions have various visual outcomes, depending on the part of the eye affected and the mechanism of injury involved. The wasp appears to have more visually disabling complications than the honey bee which has been shown to recover fully often times after treatment.[3, 4] Treatment depends on the mechanism of injury. Retained stingers are usually removed surgically [5], while tissue reactions which could be acute or chronic are treated medically [4–6]. There has been no documented report of a bee sting injury from this part of the Nigeria. This therefore becomes the first. Apart from this, report of sting in the sclera is also very rare. We report a case of ocular reactions following honeybee sting of the sclera, ocular reactions, its presumed pathogenesis, treatment and outcome.

## Patient and observation

**Figure 1:**
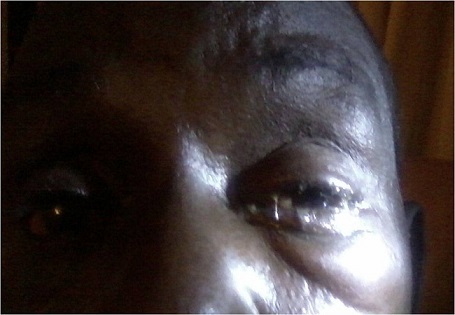
Showing mucopurulent discharge, swollen lids and mechanical ptosis

**Figure 2:**
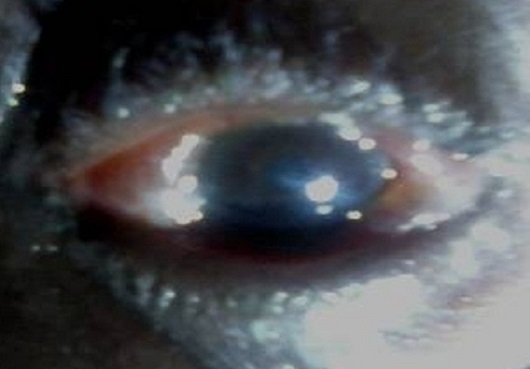
Conjunctival chemosis seen through the inter palpebral fissure

**Figure 3:**
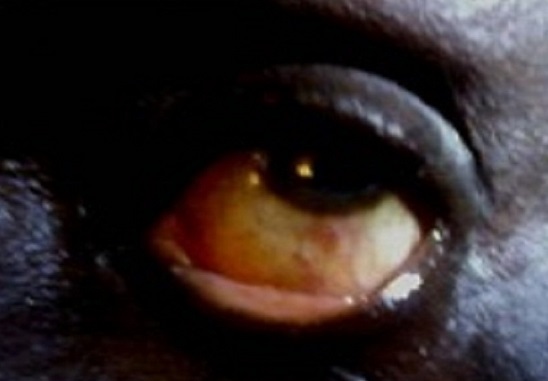
Showing stab wound site healed with staphyloma 1-1.5mm diameter and 3 to 3.5mm below the limbus at 6 O'clock

A 62 year old male farmer was attacked by a swarm of bees 2hours prior to presentation on account of severe pains. There were no previous trauma to the affected left eye or surgery, but had occasional itchy eyes. He is a known hypertensive with good control on medications, and not a known diabetic. He denied any history of harmful traditional eye medications(HTEM). Examination revealed tender swollen upper and lower lids, moderate to severe mechanical ptosis and purulent eye discharge (Figure 1). After eye toileting with normal saline, visual acuity, cornea, anterior chamber and pupillary reactions were normal but there was severe bulbar chemosis Figure 2. A swab of the discharge was sent for microscopy, culture and sensitivity (m/c/s). Treatment was thereafter instituted with Guttae Ciprofloxacin and Flurbiprofen, and oral Tramadol and NSAID. Eighteen hours after, all the symptoms had subsided. Further examination of the eye revealed a stab partial- thickness entry point of about 1 - 1.5mm in diameter in the sclera at 6 o’ clock position, 3.5 mm from the limbus and normal IOP. Dilated fundoscopy using binocular indirect ophthalmoscope, and slit lamp examination revealed normal posterior segment. There was complete resolution of the lid swelling, and chemosis within 72 hours apart from the conjunctival and episcleral hyperaemia. The purulent eye discharge stopped after 6 days and a tapered low dose oral Prednisolone was commenced. Four weeks follow up showed a self healing wound site in form of ciliary staphyloma and mild scleritis, with no evidence of erosion or irritation from a retained or migrated bee stinger (Figure 3). The result of the m/c/s showed growths of Staphylococcus aureus, and Staphyloccoccus epidermidis sensitive to ciprofloxacin, ceftazidine and erythromycin, which the topical antibiotics had taken care of.

## Discussion

This study seems to be reporting in a very long time, since 1955 by a Krennig in Germany, a case of Honeybee sting to the sclera with posterior subcapsular cataract formation. Others have reported injury to other parts of the eyes mostly from cornea and lids.[2, 4–6] The fact that the point of entry of the venom was the sclera and that the complications that developed were external and not intraocular was the interesting point in this patient.

More males are usually affected from Bee stings from previous studies because they are more exposed to this type of injury as an occupational hazard in the course of fending for their families. In Africa in particular, more men do farm work as this is culturally related. The presentation of pain, swollen upper and lower lids, and chemosis was similar to what was seen by other workers in Turkey. [7] The dissimilarity was that our subject was stung in the sclera, had purulent eye discharge as well as redness. There was no other reaction either in the cornea, anterior chamber or lens as earlier described by other authors. [1, 3] The redness and scleritis was thought to follow a reaction to inflammation of trauma and reaction to bee venom. Bee venom is known to be made up of a mixture of many substances including allergens and toxins. The process of stinging and injection of venom is known to usually present with pain and swelling. These effects could be caused by chemical mediators of anaphylaxis such as histamine, dopamine, and serotonin. Several toxins are also known to be present, including apamin, monamine, and mast-cell degranulating substances. Allergic responses could be caused by hyaluronidase and phospholipase-A2, enzymes that work to activate immune cells and produce immunoglobulin E (IgE).[8]

It's been postulated that genetic factors, together with some environmental factors could be incriminated in generating allergic reactions in some tissues. [9] Also the environment and its factors, and age may play a role in the occurrence of the acute infections. These factors coupled with the susceptibility gene could also have led to the immunologic response of the ocular tissues. The response led to the spread of venomic toxins via the episcleral tisues through the conjunctiva and the lids. Further studies are needed to establish or disprove these theories.

The lid swelling, chemosis and redness were thought to be due to combined effects of infection from micro organisms, inflammation and toxicity from the venom. The discharge yielded a growth of Staphylococcus aureus, sensitive to Ciprofloxacin and Ceftazidime. Associated purulent discharge has rarely been reported together with lid swellings. The very early tissue response of purulent discharge within 2 hours further strengthened our suspicion of an indication of the virulence of the organism and reduced host immune response of the patient. Eye toileting and broad spectrum topical antibiotics took care of this.

The presumed pathophysiology is thought to be that of a combined physical partial perforation of the sclera and the release of the venom into the adjacent sclera and episcleral tissues. This then led to the spread of the venom to the conjunctiva and skin which have receptors for antigenic reactions. There was a resultant acute allergic reaction with chemosis, conjunctivitis, blepharitis and mechanical ptosis. The delayed hypersensitivity reaction to the trauma and venom is most probably responsible for the scleritis. All resolved with anti inflammatory drugs.

Apart from surgery, topical and systemic drugs, novel methods of treating these allergies presently exist. Valovirta et al postulated that immune therapy used to target IgE-mediated allergic diseases can be administered sub lingual or sub cutaneously. However, treatment should not be given to under fives while older ones should be given titrated doses of the immune therapy.[10]

The total resolution of symptoms could be explained in part by the absence of the stinger from the tissues and the prompt treatment.

## Conclusion

Bee sting injury is not uncommon among local African farmers. Its venom can cause severe painful and inflammatory reactions to adjacent mucous membranes of the eye(conjunctiva and lid) and skin. The subject's reaction probably depends on his body's immune system, genetic constitution and virulence of the venom. Simple topical and oral anti-inflammatory drugs and mildly potent analgesics are very effective in its management. Injury can be treated without blinding complications if patients present early. Health education on early presentation and avoidance of HTEM should be promoted among the farming populations in our communities.
